# Induction of Apoptosis in Pancreatic Cancer Cells by CDDO-Me Involves Repression of Telomerase through Epigenetic Pathways

**DOI:** 10.4172/2157-2518.1000177

**Published:** 2014-05-31

**Authors:** Dorrah Deeb, Chris Brigolin, Xiaohua Gao, Yongbo Liu, Kirit R. Pindolia, Subhash C. Gautam

**Affiliations:** 1 Department of Surgery, Henry Ford Health System, Detroit, USA; 2 Department of Medical Genetics, Henry Ford Health System, Detroit, USA

**Keywords:** CDDO-Me, Apoptosis, Telomerase, hTERT, Epigenetic control

## Abstract

Reactivation of telomerase in cancers provides an attractive target for developing novel agents to selectively destroy tumor cells. Methyl-2-cyano-3,12-dioxooleana-1,9(11)-dien-28-oate (CDDO-Me), a synthetic oleanane triterpenoid, inhibited cell proliferation and induced apoptosis in pancreatic cancer cells at very low concentrations. The antiproliferative and apoptosis-inducing effects of CDDO-Me were associated with the inhibition of human telomerase reverse transcriptase (hTERT) mRNA, hTERT protein and reduction in hTERT telomerase activity. CDDO-Me inhibited multiple transcription factors that regulate hTERT expression positively (Sp1, c-Myc and NF-κB) and negatively (CTCF, E2F-1 and MAD1). CDDO-Me inhibited protein levels of DNA methyl transferases DNMT1 and DNMT3a, which also resulted in hypomethylation of hTERT promoter. In addition, transcriptionally active chromatin markers, such as acetylated histone H3 (Lys 9), acetylated histone H4, di-methyl H3 (Lys 4) and tri-methyl H3 (Lys 9) were all reduced in pancreatic cancer cells treated with CDDO-Me. Chromatin immunoprecipitation analysis showed decreased histone deacetylation and histone demethylation at hTERT promoter. Collectively, these results indicate that down-regulation of telomerase through epigenetic mechanisms plays a critical role in induction of apoptosis in pancreatic cancer cells by CDDO-Me.

## Introduction

Pancreatic ductal adenocarcinoma (PDA) is the fourth leading cause of cancer-related deaths in the United States and is almost uniformly lethal with a 5-year survival rate of < 5% [1–3]. Late initial diagnosis, aggressive metastatic behavior and resistance to chemo-radiotherapy render pancreatic cancer one of the most difficult to treat malignant diseases. Surgical resection is curative; however, nearly 80% of the patients are diagnosed with locally advanced metastatic disease, precluding surgical intervention. Gemcitabine, the current standard of care for advanced pancreatic cancer, provides short-term symptomatic improvement with minor impact on survival and integration of multiple modalities has not improved survival [4,5]. Thus, there is a dire need to search for more active agents and novel strategies to treat pancreatic cancer.

Telomeres are nucleoprotein structures present at the end of chromosomes, which are essential in maintaining chromosome stability and integrity by preventing end-to-end fusion and chromosomal rearrangement [6]. During each cell division, telomere length is progressively shortened due to gradual loss of telomeric DNA repeat sequence (TTAGGG) [7,8]. The shortening of telomeres beyond a critical threshold leads to replicative senescence or apoptosis. Telomerase, a reverse transcriptase maintains the telomere length by adding the hexameric DNA repeats (TTAGGG) to the 3′ flanking end of DNA strands in telomeres. The human telomerase complex consists of telomerase reverse transcriptase (hTERT), telomerase RNA template (TERC), telomerase associated protein-1 (TEP-1), hsp90 and p23 [9–11]. The telomerase activity in humans is highly regulated and is detectable only in germ line cells and some stem cells but is repressed in somatic cells [12,13]. Deregulated telomerase activity is associated with promotion of tumorigenesis and neoplastic growth of cancers [7,14,15]. In fact, approximately 90% of human cancers including pancreatic cancer exhibit reactivation of telomerase activity, contributing to the unlimited proliferation and replicative potential of cancer cells [11,16]. Thus, cancer-specific activation of telomerase provides an attractive target for selectively killing cancer cells with novel agents without damaging normal cells. Indeed, we have recently shown that inhibition of cell proliferation and induction of apoptosis in pancreatic cancer cells by methyl-2-cyano-3,12-dioxooleana-1, 9(11)-dien-28-oate (CDDO-Me), a synthetic oleanane triterpenoid, is associated with the repression of hTERT expression, the gene that codes for telomerase, and telomerase activity [17]. However in that study, experiments were performed using high concentrations of CDDO-Me and the mechanism of inhibition of hTERT expression was not adequately investigated. In the present study, we investigated the anti-proliferative and apoptosis-inducing activity of CDDO-Me in pancreatic cancer cells at very low concentrations and the effect they have on epigenetic regulatory processes involved in hTERT expression.

## Materials and Methods

### Reagents

CDDO-Me was obtained from the National Cancer Institute, Bethesda, MD through the Rapid Access to Intervention Development Program. A 100 mM stock solution of CDDO-Me was prepared in DMSO, which was subsequently diluted in tissue culture medium to obtain the working concentrations. Antibodies against PARP-1, NF-κB (p65), Sp1, c-Myc and β-actin were purchased from Santa Cruz Biotechnology, Inc. (Santa Cruz, CA). Anti-hTERT and p-TERT (Ser^824^) antibodies were obtained from-Abcam Inc. (Cambridge, MA). Antibodies against DNMT1 and DNTM3α were from Cell Signaling (Danvers, MA). Anti-acetylated histone H3 at lysine 9 (ac-H3K9), anti- acetylated histone H4 (ac-H4), anti-histone dimethyl-H3 lysine 4 (di-me-H3K4) and anti-trimethy-H3 lysine 9 (ac-tri-me-H3K9) were purchased from Millipore (Temecula, CA). Annexin V-FITC apoptosis detection kit II was obtained from BD Pharmingen (San Diego, CA, USA) and TRAPeze telomerase detection kit was purchased from Millipore (Millipore, Temecula, CA).

### Cell lines

Human pancreatic cancer cell lines MiaPaCa-2 and Panc-1 were obtained from the American Type Culture Collection (ATCC), Rockville, MD, USA. Both cell lines were cultured in DMEM tissue culture medium (Gibco BRL, Rockville, MD) supplemented with 10% fetal bovine serum, 1% penicillin/streptomycin, and 25 mM HEPES buffer at 37° C in a humidified atmosphere consisting of 5% CO_2_ and 95% air. Cells were maintained by splitting cultures twice a week.

### Measurement of cell viability

0.5×10^6^ Panc-1 or MiaPaCa-2 pancreatic cancer cells in 10 mL tissue culture medium were added to 100 mm^2^ petri plates and allowed to adhere for 24 h. Cells were then treated with CDDO-Me at concentrations ranging from 0 to 0.5 μM for 5 days in triplicates. At the end of incubation period, cells were harvested by trypsinization and viability determined by trypan blue dye exclusion using a hemocytometer.

### Apoptosis assay

Apoptosis was assessed by the binding of annexin V-FITC to phosphotidylserine, which is externalized to the outer leaflet of the plasma membrane early during induction of apoptosis. Briefly, untreated cells and cells treated with CDDO-Me were resuspended in the binding buffer provided in the annexin V-FITC apoptosis detection kit II (BD Biosciences, San Diego, CA, USA) and allowed to react with 5 μl of annexin V-FITC reagent and 5 μl of propidium iodide (PI) for 30 min at room temperature in the dark. Stained cells were analyzed by flow cytometry using Accuri C6 flow cytometer (Accuri Cytometers Inc. Ann Arbor, MI). The induction of apoptosis by CDDO-Me was confirmed from the cleavage of PARP-1 by western blotting.

### Measurement of hTERT expression

The effect of CDDO-Me on hTERT expression was measured by analyzing hTERT mRNA and hTERT protein. For hTERT mRNA, total cellular RNA was extracted with TRI-zol reagent (GIBCO) according to the manufacturer’s recommendation. 1 μg of RNA was then reverse transcribed by oligo-dt primer and high fidelity reverse transcriptase (Boehringer Mannheim, Germany) to generate cDNAs. One μL of cDNA was used as the template for polymerase chain reaction (PCR) using hTERT primers: upper, 5′-TGTTTCTGGATTTGCAGGTG-3′, and lower, 5′-GTTCTTGGCTTTCAGGATGG-3′; and GAPDH primers: upper, 5′-TCCCTCAAG ATTGTCAGCAA-3′, and lower, 5′-AGATCCACAACGGATACATT-3′. The PCR conditions used were 33 cycles of denaturation (95°C for 1 min), annealing (62°C for 30 sec) and polymerization (72°C for 1 min). The PCR products were separated on 2% agarose gel electrophoresis and visualized by ethidium bromide staining. Gels were photographed and band densities were analyzed using the NIH/Scion image analysis software. The hTERT primers amplified a DNA fragment of 200 bp and the DNA fragment size amplified by GAPDH primers was 173 bp.

Total and phosphorylated hTERT protein levels were measured by western blotting as described below.

### Western blotting

Cell lysates were prepared in lysis buffer containing 1% Triton-X 100 (v/v), 10 mM Tris-HCl (pH 7.5), 5 mM EDTA, 150 mM NaCl, 10% glycerol, 2 mM sodium vanadate, 5 μg/mL leupeptin, 1 μg/mL aprotinin, 1 μg/mL pepstatinin, and 10 μg/mL 4-2-aminoethyl-benzenesulfinyl fluoride). Lysates were clarified by centrifugation at 14,000 x g for 10 min at 4°C, and protein concentrations were determined by Bradford assay. Samples (50 μg) were boiled in an equal volume of sample buffer (20% glycerol, 4% SDS, 0.2% Bromophenol Blue, 125 mM Tris-HCl (pH 7.5), and 640 mM 2-mercaptoethanol) and separated on pre-casted Tris-glycine polyacrylamide gels using the XCell SurelockTM Mini-Cell, in Tris-Glycine SDS running buffer, all from Novex (Invitrogen, Carlsbad, CA). Proteins resolved on the gels were transferred to nitrocellulose membranes. Membranes were blocked with 5% milk in 10 mM Tris-HCl (pH 8.0), 150 mM NaCl with 0.05% Tween 20 (TPBS) and probed using target specific antibodies or β-actin as loading control and HRP-conjugated secondary antibody. Immune complexes were visualized with enhanced chemiluminescence. Protein bands were imaged and band densities analyzed by NIH/Scion image analysis software. The protein band densities were normalized to the corresponding β-actin band densities.

### Telomerase activity assay

The telomerase activity in cell extracts was assessed by the PCR-based telomeric repeat amplification protocol (TRAP) using TRAPeze gel-based telomerase detection kit (Millipore, Temecula, CA). Briefly, cells were extracted in CHAP lysis buffer on ice for 30 minutes. Two μL (100 ng) of cell extract was added to the TRAP reaction mixture containing dNTPs, TS primer, TRAP primers and Taq polymerase and incubated at 30°C for 30 minutes in a thermocycler followed by 3-step PCR at 94°C/30 sec, 59°C/30 sec, and 72°C/1 minute for 33 cycles. The PCR products were fractionated on nondenaturing 12.5% polyacrilamide gel and visualized by staining with ethidium bromide. The ladder of products with 6 base pair increment indicating telomerase activity was analyzed with NIH/Scion image analysis software. The cumulative band density for each lane was normalized to the corresponding band density of internal control (36 bp).

### Chomatin immunoprecipitaion (ChIP) assay

ChIP analysis of transcriptionally active chromatin markers interacting with hTERT promoter was performed using the EZ-ChIP kit (Upstate Biotechnology) according to the instructions included in the kit. ChIP-validated antibodies used were: anti-acetyl-histone H3 lysine 9, anti-acetyl-histone H4, anti-tri-methyl histone H3 lysine 9 and anti-di-methyl histone H3 lysine 4, all from Millipore. ChIP-purified DNA from control cells (untreated) and cells treated with CDDO-Me (0.125–0.5 μM) for 5 days was amplified by PCR using hTERT promoter primers: forward, 5′-TCCCCTTCACGTCCGGCATT-3′; reverse, 5′-AGCGGAGAGAGGTCGAATCG-3′. The PCR products were separated on 2% agarose gel electrophoresis and visualized by ethidium bromide staining. The hTERT primers amplified a DNA fragment of 200 bp.

### Bisulfite sequencing analysis

Genomic DNA from Panc-1 cells treated or not with CDDO-Me for 5 days was isolated using Blood and Cell Culture DNA mini kit (Qiagen, Valencia, CA). 2 μg of DNA was used in an overnight bisulfite reaction using the Epitect Bisulfite Conversion kit (Qiagen, Valencia, CA) according to the instruction provided with the kit.

Following the genome wide conversion of unmethylated Cs to Ts, 0.1–0.2 μg of DNA from each sample was used in a 20μl PCR reaction carried out at 95°C for 5 minutes for initial denaturation followed by 40 cycles of [95°C for 30 seconds, 62°C for 45 seconds, and 72°C for 60 seconds], and final extension step of 72°C for 10 minutes. The bisulfite specific primers were designed using Methyl Primer Express software from ABI (LifeTechnology, Grand Island, NY). The primers were designed to non-discriminately amplify hTERT promoter region spanning −248 to +108. The forward primer sequence was 5′-GTTTTTTAYGTGGYGGAG-3′ and the reverse primer sequence was 5′-CCACCCTAAAACCCCAA-3′. PCR products were fractionated on 1.7% agarose gel and 356bp PCR fragments were cut out from the agarose gel and purified using QIAQuick Gel Extraction kit (Qiagen Valencia, CA). The purified PCR products were cloned using TA cloning kit (LifeTechnology, Grand Island, NY). Positive clones confirmed by restriction digestion were sequenced (Sequencing Core, University of Michigan, Ann Arbor, MI).

### Statistical analysis

Most data are presented as means ± S.D. Outcomes for treated and untreated cells were compared by Student’s t-test. Differences were considered significant at p<0.05.

## Results

### CDDO-Me inhibits proliferation and induce apoptosis in pancreatic cancer cells

**Figure 1:**
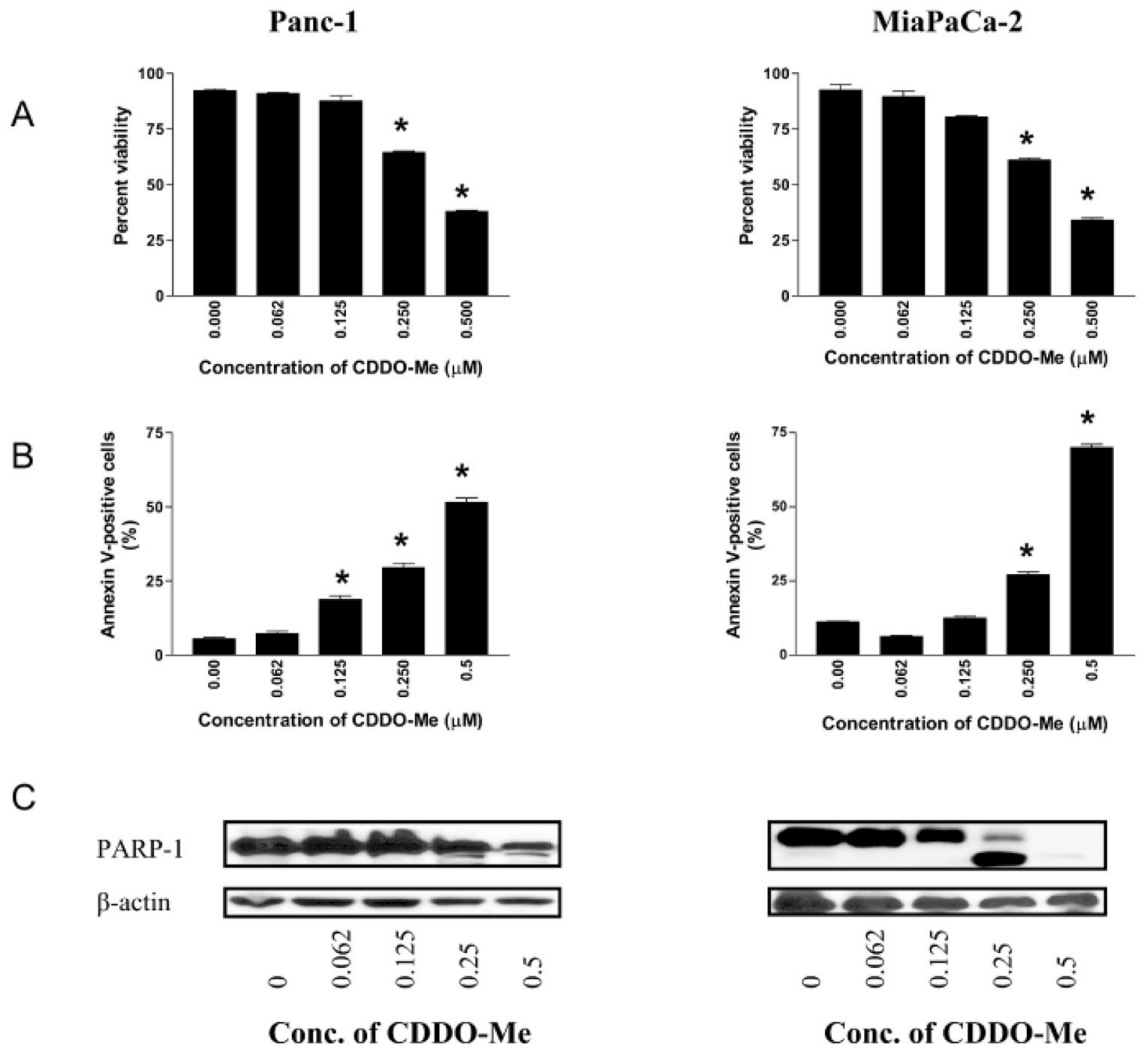
CDDO-Me induces apoptosis in pancreatic cancer cells A. Panc-1 and MiaPaCa-2 pancreatic cancer cells (0.5×10^6^/dish) were treated with CDDO-Me at concentrations ranging from 0 to 0.5 μM for 5 days in triplicates. At the end of incubation period, cells were harvested and viability determined by trypan blue dye exclusion using hemocytometer. B. Annexin V-FITC binding. Tumor cells were treated with CDDO-Me at 0 to 0.5 μM for 5 days and then reacted with 5 μl of annexin V-FITC and 5 μl PI for 30 min and the percentage of annexin V-FITC binding cells was determined by flow cytometry. C. Cleavage of PARP-1 in cells treated with CDDO-Me was analyzed by immunoblotting. Similar results were obtained in 3 independent experiments. *P<05 compared to control cells (no CDDO-Me).

To measure the effect of low concentrations of CDDO-Me on proliferation, Panc-1 and MiaPaCa-2 cells were treated with CDDO-Me at concentrations ranging from 0.125 to 0.5 μM for five days. At the end of the treatment, viability of cultures was determined by counting cells stained with trypan blue using a hemocytometer. As shown in Figure 1A, CDDO-Me significantly reduced the proliferation of both cell lines (measured from the loss of viability of cultures) at concentrations of 0.125 μM to 0.5 μM (p<0.05).

To determine whether CDDO-Me induces apoptosis in pancreatic cancer cells, we first measured the binding of annexin V-FITC to Panc-1 and MiaPaCa-2 cells treated with CDDO-Me by flow cytometry. Since cells in early-stage apoptosis are stained by annexin V-FITC only and those in advanced stages of apoptosis are stained both by annexin V-FITC and PI, data presented in Figure 1B are average of cells stained by annexin V-FITC only plus cells dually stained by annexin V-FITC and PI (see supplementary Figure S1). Treatment with CDDO-Me (0.125 to 0.5 μM) significantly increased the percentage of annexin V-FITC plus annexin V-FITC/PI binding cells in both cell lines [Panc-1 cell, 19% to 52% at 0.125 to 0.5μM CDDO-Me (p<0.05); MiaPaCa-2 cells, 13% to 69% at 0.125 to 0.5 μM CDDO-Me (p<0.05)].

The induction of apoptosis by CDDO-ME was confirmed by the cleavage of PARP-1 by western blotting. As shown in Figure 1C, native PARP-1 (110 kDa) was clearly cleaved in MiaPaCa-2 cells at CDDO-Me concentrations of 0.125 to 0.5 μM as identified by reduction in total PARP-1 levels and by the appearance of a cleaved PARP-1 fragment (89 kDa). Although native PARP-1 was also reduced in Panc-1 cells at 0.25–0.5 μM CDDO-Me, but the cleaved PARP-1 fragment was only weakly detectable.

### CDDO-Me inhibits expression of hTERT gene in pancreatic cancer cells

**Figure 2:**
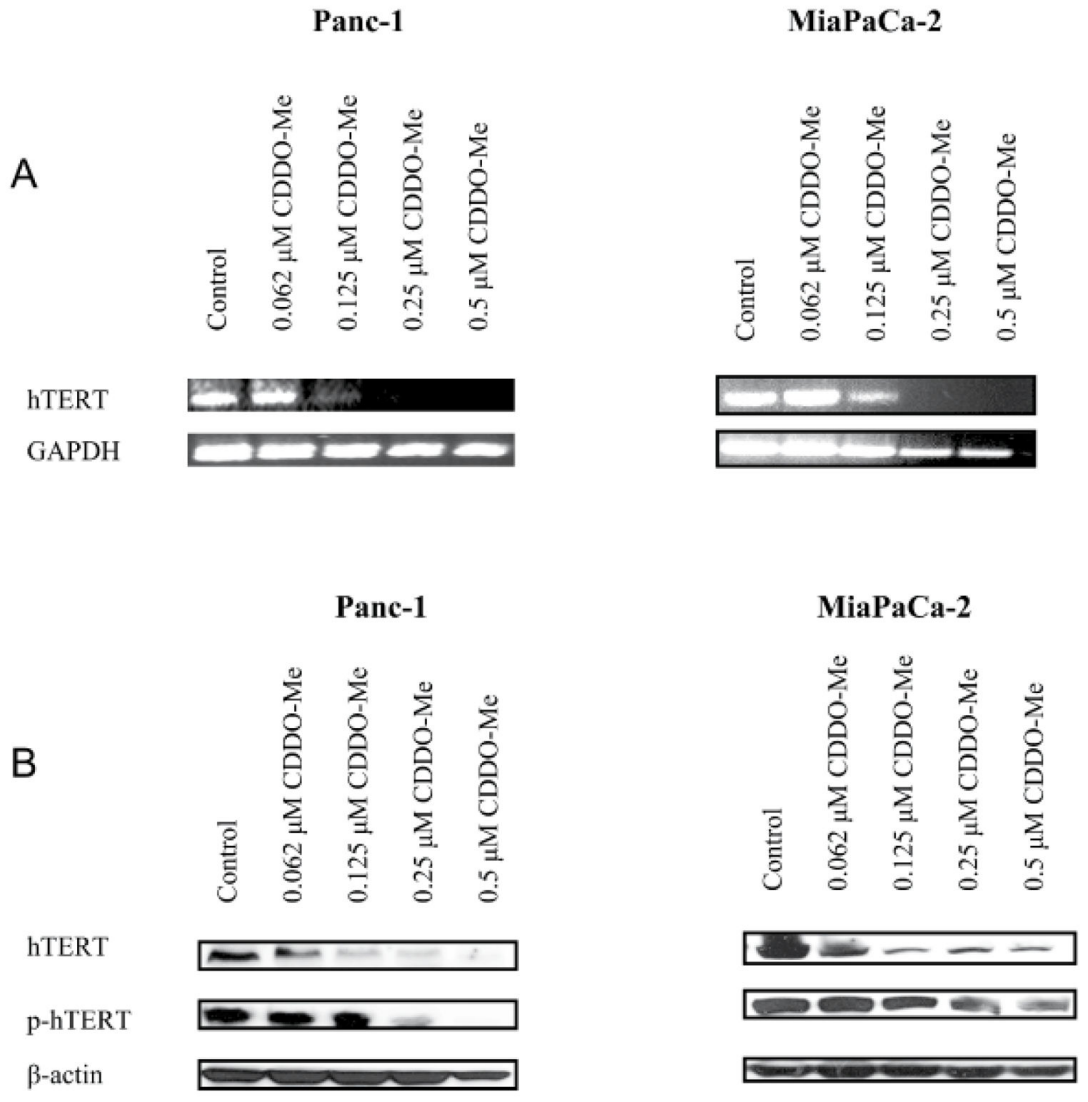
CDDO-Me inhibits hTERT expression in pancreatic cancer cells A. Effect of CDDO-Me on hTERT gene expression. Panc-1 and Mia PaCa-2 cells were treated with CDDO-Me (0–0.5 μM) for 5 days and total cellular RNA was prepared using TRI-zole reagent. 1 μg of cellular RNA was reverse transcribed using oligo-dt primer and high fidelity reverse transcriptase. 1 μL of cDNA was amplified using hTERT or GAPDH primers. Amplified products were separated on 2% DNA agarose gel. Gels were stained with ethidium bromide and DNA fragments were identified by base pair size. B. Effect of CDDO-Me on hTERT protein. Panc-1 and MiaPaCa-2 cells were treated with CDDO-Me as described above and cell lysates were analyzed for hTERT and p-hTERT protein by western blotting. Each experiment was repeated at least three times.

The inhibition of telomerase leads to cellular senescence and/or apoptosis [18,19]. Thus, we determined the effect of CDDO-Me on the expression hTERT and hTERT telomerase activity. The effect of CDDO-Me on hTERT expression was measured by analyzing hTERT mRNA and hTERT protein expression. Analysis of hTERT mRNA by RT-PCR showed more than 50% inhibition of hTERT mRNA in both cell lines after treatment with CDDO-Me at 0.125 μM for 5 days. Complete inhibition of hTERT mRNA was observed at 0.25–0.5 μM CDDO-Me without significantly affecting the expression of GAPDH in Panc-1 cells, but GAPDH mRNA in MiaPaCa-2 cells was partially reduced at 25–0.50 μM CDDO-Me (Figure 2A). CDDO-Me also inhibited the levels of native hTERT protein at 0.062–0.5 μM in both cell lines (Figure 2B). Since phosphorylation of the catalytic subunit of hTERT is necessary for its telomerase activity, we also measured the effect of CDDO-Me on phosphorylated hTERT. As shown in Figure 2B, CDDO-Me also inhibited p-hTERT at concentrations of 0.25–0.5 μM (Figure 2B).

Whether inhibition of hTERT expression by CDDO-Me results in decrease in telomerase activity was investigated next. After treatment with CDDO-Me (0.062 to 0.5 μM) for 5 days, Panc-1 and MiaPaCa-2 cells were extracted in CHAP lysis buffer and the telomerase activity in extracts was measured by the PCR-based TRAP assay. Treatment with CDDO-Me drastically reduced the telomerase activity in a dose-dependent manner, resulting in 90%–100% reduction in telomerase activity in both cell lines (Figure S2).

Collectively, attenuation of hTERT mRNA, basal and phospho-hTERT protein and telomerase activity by CDDO-Me indicated that telomerase is a potential target of CDDO-Me in pancreatic cancer cells.

### CDDO-Me inhibits transcription factors that regulate hTERT expression

**Figure 3:**
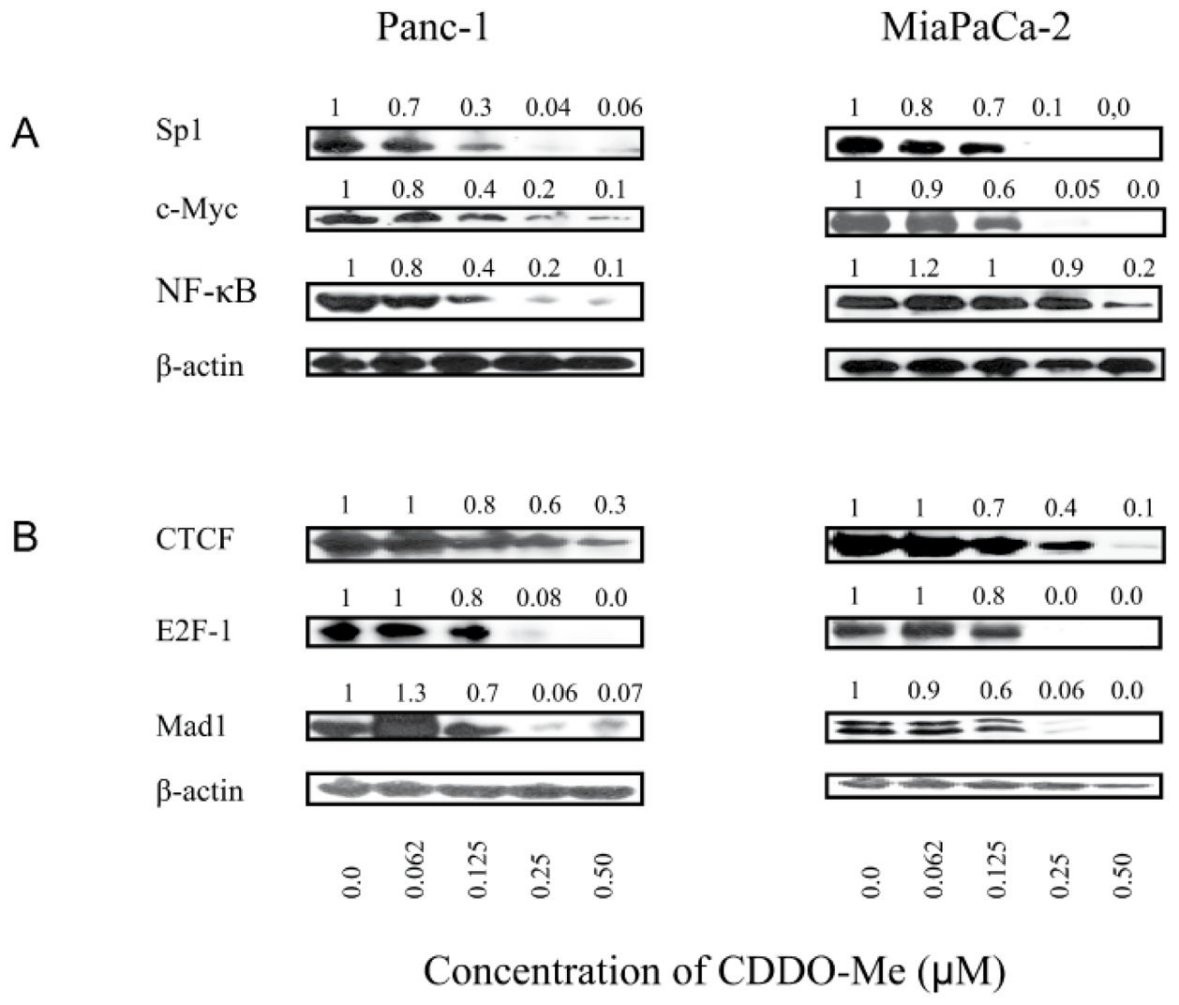
Effect of CDDO-Me on transcription factors that regulate hTERT expression Panc-1 and MiaPaCa-2 cells were treated with CDDO-Me (0–0.5 μM) for 5 days and cell lysates were analyzed for transcription factors that regulate hTERT expression positively (A) or negatively (B) by western blotting. This experiment was repeated three times.

The transcription of hTERT gene is regulated by a number of transcription factors. The hTERT core promoter contains transcription factor binding sites for Sp1, c-Myc, NF-κB and STAT-3 [20–22] that up-regulate hTERT expression. Therefore, we assessed the effect of CDDO-Me on the levels of these proteins. Treatment with CDDO-Me (0 to 0.5 μM) for 5 days partially to completely reduced the levels of Sp1, c-Myc and NF-κB (p65) at 0.125 to 0.5 μM CDDO-Me (Figure 3A), suggesting that inhibition of these transcription factors likely contributes to the inhibition of hTERT transcription by CDDO-Me.

hTERT expression is also regulated by transcription factors that repress hTERT expression (e.g., CTCF, E2F-1 and MAD-1), therefore we analyzed the effect of CDDO-Me on these proteins. As shown in Figure 3B, contrary to our expectations CDDO-Me also decreased these proteins at 0.125 to 0.5 μM in both cell lines. Thus, CDDO-Me inhibits both the up-regulators (Sp1, c-Myc and NF-κB) and repressors (CTCF, E2F-1 and MAD-1) of hTERT gene transcription.

### Effect of CDDO-Me on epigenetic regulation of hTERT expression

**Figure 4:**
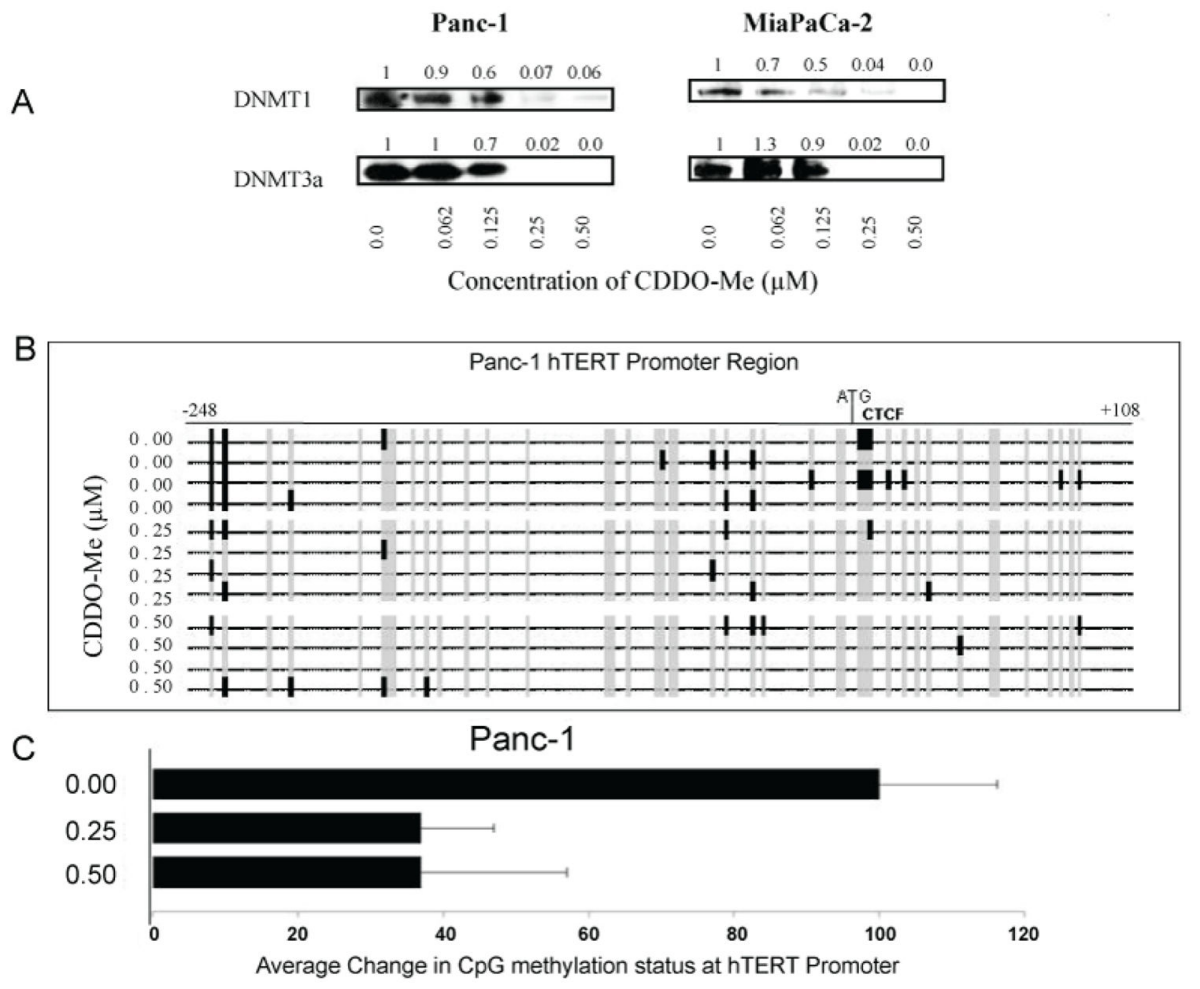
Treatment with CDDO-Me alters methyl transferases and methylation status of hTERT promoter A. Panc-1 and MiaPaCa-2 cells were untreated or treated with CDDO-Me (00–0.5 μM) for 5 days and cellular levels of DNMT1 and DNMT3a DNA methyl transferases were measured by western blotting. B. Methylation status of CpGs at hTERT promoter 5′ transcription regulatory and intergenic transcription regulatory region (248 to +108 nucleotides) in Panc-1 cells was assessed by bisulfite sequencing as described in Materials and Methods. C. Data are presented as average percent change in methylated CpG sites in the hTERT regulatory region after treatment with CDDO-Me.

hTERT gene expression is epigenetically-regulated through promoter methylation and histone modifications [23]. Whether hTERT suppression by CDDO-Me involved modulation of the epigenetic pathways of hTERT gene expression was evaluated. First, we analyzed the effect of CDDO-Me on DNA methyltransferases in Panc-1 and MiaPaCa-2 cells treated with CDDO-Me. CDDO-Me caused a concentration-dependent decrease in DNA methyltransferases DNMT1 and DNTM3α in both cell lines (Figure 4A). Next, we examined the methylation status of hTERT promoter in Panc-1 cells treated with CDDO-Me at two different concentrations as shown in Figures 4B and 4C. Out of 43 putative CpGs at hTERT promoter region analyzed in Panc-1 cells, untreated cells on average showed 7 methylated CpGs whereas the cells treated with CDDO-Me at 0.25 μM and 0.5 μM on average showed 3 methylated CpGs. Most of these changes were found surrounding the translational start site (ATG) and chromatin domain control region (CTCF).

**Figure 5:**
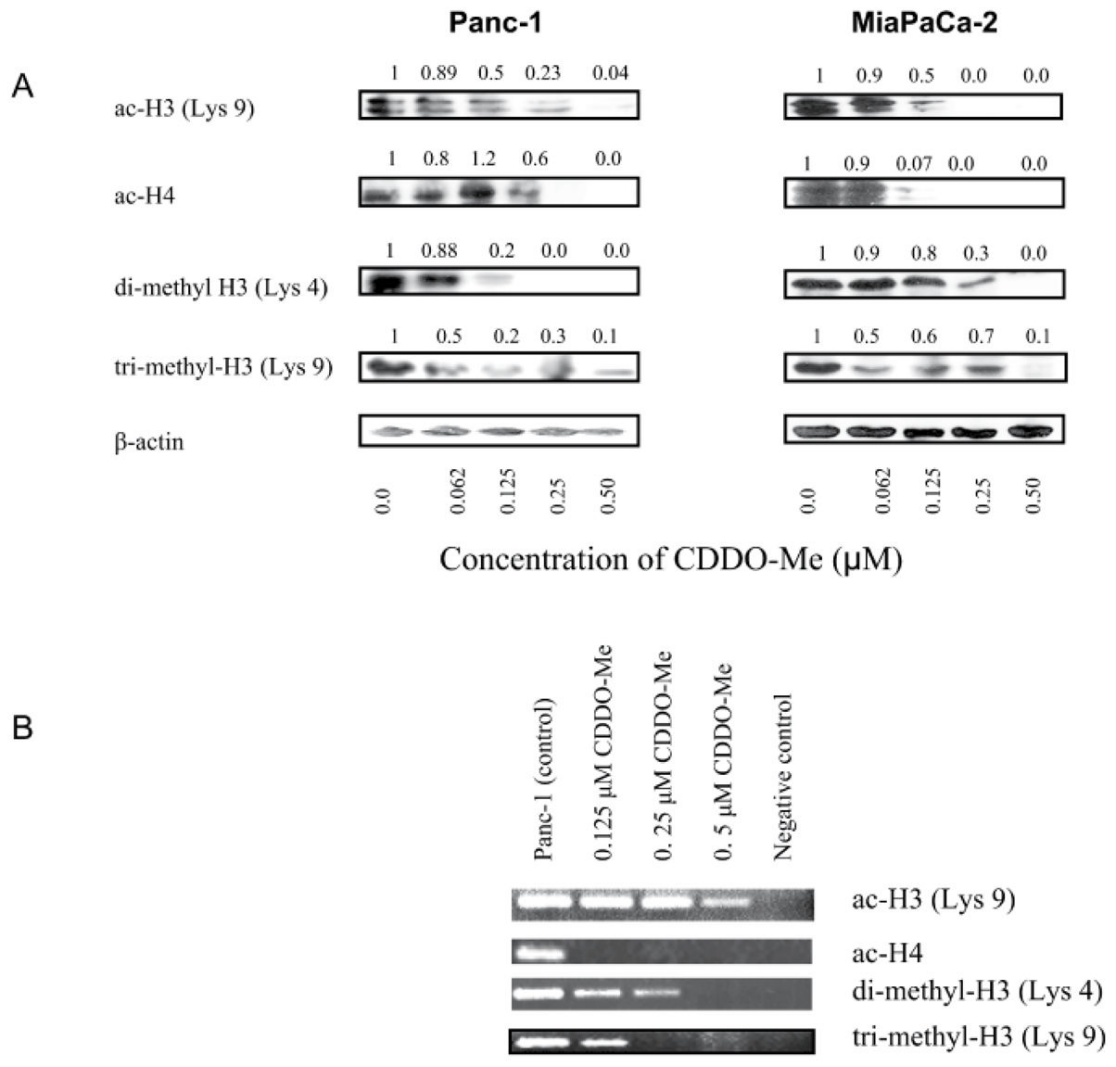
CDDO-Me induces chromatin modifications. A. Effect on transcriptionally active chromatin markers. Panc-1 and MiaPaCa-2 cells were treated with CDDO-Me (0–0.5 μM) for 5 days and cell lysates were analyzed for acetylated histone H3 (Lys 9), acetylated histone H4, di-methyl H3 (Lys 4) and tri-methyl H3 (Lys 9) by western blotting. B. CDDO-Me induces changes in histone modification in regulatory region of hTERT promoter. Panca-1 cells were treated with CDDO-Me as described above and active chromatin markers interacting with hTERT promoter (acetylated histone H3 (Lys 9), acetylated histone H4, di-methyl H3 (Lys 4) and tri-methyl H3 (Lys 9) were analyzed by ChIP-PCR assay as described in Materials and Methods. No antibody controls were included to verify ChIP efficacy. The experiment was repeated two times.

In addition to DNA methylation, histone acetylation and histone methylation play pivotal roles in hTERT expression. Whether treatment with CDDO-Me affects histone modifications was investigated next. For this, we first examined the effect of CDDO-Me on cellular levels of transcriptionally active acetylated histone H3 at lysine 9 (ac-H3K9) and acetylated histone H4 (ac-H4, Figure 5A). Treatment with CDDO-Me dose-dependently reduced the levels of ac-H3K9 and ac-H4 in both cell lines (Figure 5A). Similarly, levels of histone markers dimethyl-H3 lysine 4 (di-me-H3K4) and trimethy-H3 lysine 9 (ac-tri-me-H3K9) were also reduced in cells treated with CDDO-Me (Figure 5A).

Decrease in transcriptionally active histone markers described above suggested that attenuation of hTERT expression by CDDO-Me might result from changes in histone modification in the hTERT promoter. Thus, we analyzed changes in histone acetylation and histone methylation in the regulatory region of hTERT promoter by ChIP assay in Panc-1 cells treated with CDDO-Me. As shown in Figure 5B, acetylated histone H3 at lysine 9 was reduced in cells treated with CDDO-Me at 0.5 μM, whereas acetylated histone H4 was completely inhibited even at the lowest concentration of 0.125 μM. Histone methylation histone markers dimethyl-H3 lysine 4 and trimethy-H3 lysine 9 were also decreased in cells treated with CDDO-Me at concentrations of 0.0625 to 0.5 μM. These data indicated that inhibition of hTERT expression by CDDO-Me involves inhibition of histone modifications in hTERT regulatory region.

## Discussion

CDDO-Me is a multifunctional compound with potent anti-inflammatory and anticarcinogenic activity [24]. CDDO-Me inhibits proliferation and induces apoptisis in diverse cancer cell types in cell culture [25–28] and inhibit the growth of tumor implants and prevent development of cancers in mouse models [29–31]. The anticarcinogenic mechanisms of CDDO-Me involves inhibiting a number of prosurvival signaling pathways, such as MAPK (Erk1/2), NF-κB, and Akt/mTOR signaling [32–34]. hTERT expression and telomerase activity is elevated in vast majority of cancers including pancreatic cancers [11,16,35–37]. Reexpression of telomerase provides unlimited proliferative advantage to cancer cells and telomerase inhibition inhibits cell proliferation, inducing cellular senescence or apoptosis. Little is known about the interplay between the anticancer mechanisms of CDDO-Me and telomerase. In a previous study we have shown that inhibition of cell proliferation and induction of apoptosis in pancreatic cancer cells by CDDO-Me is associated with the inhibition of hTERT gene that codes for the catalytic subunit of telomerase and its telomerase activity; however, the molecular mechanism of hTERT inhibition by CDDO-Me was not elucidated. The results of the present study confirm our previous findings that induction of apoptosis even at very low concentrations of CDDO-Me used in this study is associated with the inhibition of hTERT expression and its telomerase activity. CDDO-Me could repress hTERT by inhibiting hTERT gene transcription and/or hTERT protein production. Our data showed inhibition of both hTERT gene expression and protein production. Further, it also inhibited the phosphorylation of hTERT protein. The attenuation of hTERT mRNA, basal hTERT and phospho-hTERT suggested that CDDO-Me might also inhibit telomerase activity. Indeed, our data demonstrated that treatment with CDDO-Me reduced the telomerase activity in both pancreatic cancer cell lines (Figure S2). Although reduction in cellular telomerase activity can be attributed to the inhibition of hTERT gene expression and/or inhibition of phosphorylation of hTERT, these data do not demonstrate whether CDDO-Me is able to directly inhibit the telomerase activity of hTERT. Overall, attenuation of hTERT gene expression, hTERT protein production and phosphorylation and telomerase activity indicated that inhibition of telomerase is part of the mechanism by which CDDO-Me inhibits proliferation and induce apoptosis in pancreatic cancer cells. These findings are in agreement with other reports showing that inhibition of hTERT telomerase activity is necessary for the antiproliferative and apoptosis-inducing activity of natural compounds including genistein, sulforaphane and green tea polyphenols [38–40]. However, more work is required to determine whether CDDO-Me directly binds and degrades RNA template of telomerase and if it also causes shortening of telomeres.

A number of factors and molecules that regulate hTERT transcription have been identified. The hTERT core promoter contains binding sites for several transcription factors such as Sp1, c-Myc and NF-κB and STAT-3 [20–22]. Inhibition of these transcription factors would likely impact transcription of hTERT gene. Indeed, we found that CDDO-Me inhibited Sp1, c-Myc and NF-κB in Panc-1 and MiaPaCa-2 cells, indicating that diminished hTERT expression and protein production by CDDO-Me may be attributed to the inhibition of these transcription factors. Contrary to our expectations however, various repressors of gene transcription, such as CTCF, E2F-1 and MAD1 that negatively regulate hTERT expression were also reduced in cells treated with CDDO-Me. Since CDDO-Me inhibited transcription factors that both up-regulate and down-regulate hTERT gene expression, how is hTERT gene expression then inhibited? One possibility is that CDDO-Me exerts more inhibitory function on transcription factors that up-regulate hTERT expression (Sp1, c-Myc and NF-κB and STAT-3) than those that down-regulate its expression (e.g., CTCF, E2F-1 and MAD1). This conclusion however requires further elucidation.

As stated before, epigenetic mechanisms play critical roles in regulating hTERT expression. Contrary to the prevalent view that hypermethylation of gene promoters typically inhibits their transcription; hypermethylation of hTERT promoter is associated with increased hTERT expression [41,42]. Epigenetically, genes expression can be regulated through processes such as DNA methylation, chromatin remodeling and modulation of the activity of enzymes and factors associated with these processes. Studies have shown that DNA methylation plays an important role in hTERT transcription and DNA methylation is primarily the function of DNMTs [43]. DNMT1, a maintenance methyltransferase, maintains hypermethylation of hTERT promoter, whereas DNMT3a and DNMT3b are responsible for *de novo* activity. Treatment with CDDO-Me inhibited DNMT1 and DNMT3a in Panc-1 and MiaPaCa-2 cells. As expected, the inhibition of DNMT1 resulted in demethylation of hTERT promoter. The number of methylated CpGs in hTERT promoter was significantly reduced following treatment with CDDO-Me. These data correlated with the inhibition of hTERT expression and suggest that promoter demethylation plays an important role in inhibition of hTERT expression by CDDO-Me. Demethylation of hTERT promoter allows binding of repressors, such as CTCF or E2F-1 and silencing of hTERT expression [39,40]. CDDO-Me not only caused demethylation of hTERT promoter but also suppressed CTCF, E2F-1 and MAD-1. Thus, the exact mechanism by which demethylation of hTERT promoter leads to the inhibition of hTERT expression by CDDO-Me remains elusive.

Besides DNA methylation, histone acetylation and methylation also play critical roles in hTERT expression [44]. Histone modifications result in loosening of the chromatin, allowing binding of the activators and/or repressors of gene transcription at the gene promoters. We found decrease in cellular levels of transcriptionally active chromatin markers acetylated histones H3 and H4. CDDO-Me also affected the methylation of histone, since di-methyl-H3 lysine 4 and trimethyl-H3K9 were also reduced in cells treated with CDDO-Me. The alterations in chromatin markers were also found at the hTERT promoter. ChIP analysis showed decrease in ac-H3, ac-H4, dimethyl-H3 and tri-methy-H3K9 at hTERT promoter in cells treated with CDDO-Me. Together, these data demonstrate that inhibition of epigenetic processes such as DNA methylation and chromatin modifications plays a crucial role in inhibition of hTERT expression by CDDO-Me in pancreatic cancer cells. These findings corroborate the results of other studies in which other anticancer agents also inhibited hTERT expression in tumor cells by interfering with the epigenetic regulatory processes [23,38–40].

## Conclusion

The findings presented in this paper demonstrated that induction of apoptosis in pancreatic cancer cells by CDDO-Me is associated with the inhibition of hTERT and its telomerase activity. CDDO-Me inhibited hTERT mRNA and transcription factors that regulate hTERT gene expression positively and negatively (Sp1, c-Myc, NF-κB, CTCF, E2F-1 and MAD-1). Among the epigenetic pathways of gene regulation, CDDO-Me inhibited, hTERT promoter methylation, DNA methytransferases and histone modifications (acetylation and methylation). Together, these data indicated that modulation of epigenetic processes plays a critical role in inhibition of telomerase in pancreatic cancer cells by CDDO-Me.

## Supplementary Material

Fig. S1

Fig. S2
