# Supplementary material for: Induction of Apoptosis in Pancreatic Cancer Cells by CDDO-Me Involves Repression of Telomerase through Epigenetic Pathways
Source: J Carcinog Mutagen. Author manuscript; Available in PMC 2014 Aug 20. (PMC4139055; doi:10.4172/2157-2518.1000177)
Supplement: Fig. S1 [file NIHMS613814-supplement-Fig__S1.pdf]

Panc-1

MiaPaCa-2

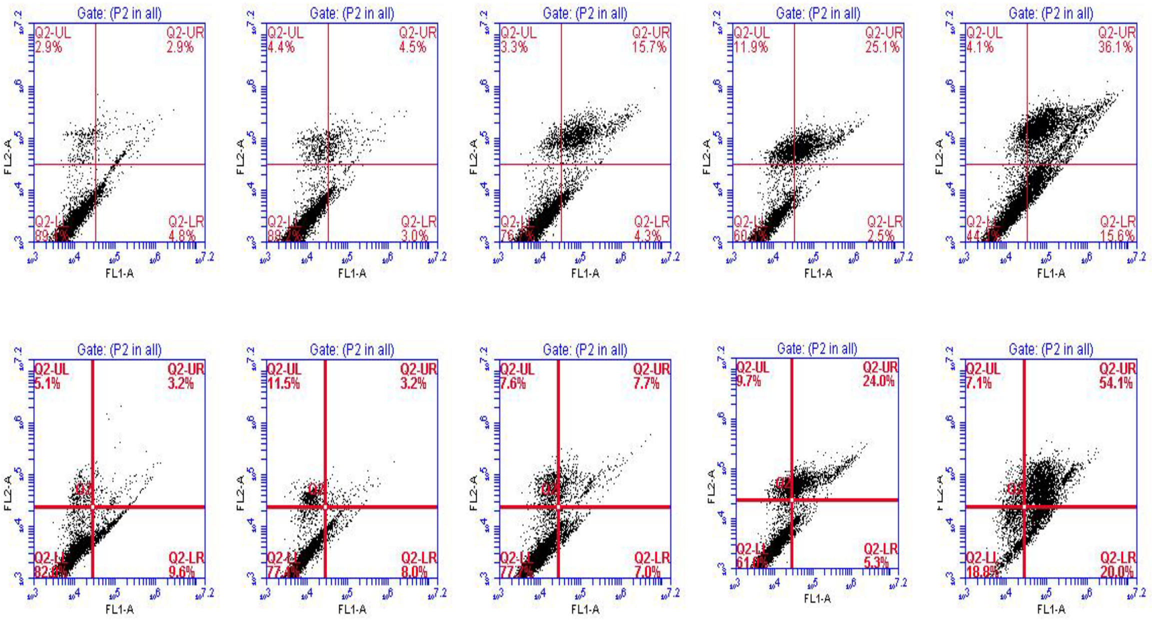

0.0 0.062 0.125 0.25 0.5

Concentration of CDDO-Me (μM)

Fig. S1. Flow cytographs showing annexin V-FITC/PI staining.
