# Supplementary figures and images for: Induction of Apoptosis in Pancreatic Cancer Cells by CDDO-Me Involves Repression of Telomerase through Epigenetic Pathways
Source: J Carcinog Mutagen. Author manuscript; Available in PMC 2014 Aug 20. (PMC4139055; doi:10.4172/2157-2518.1000177)

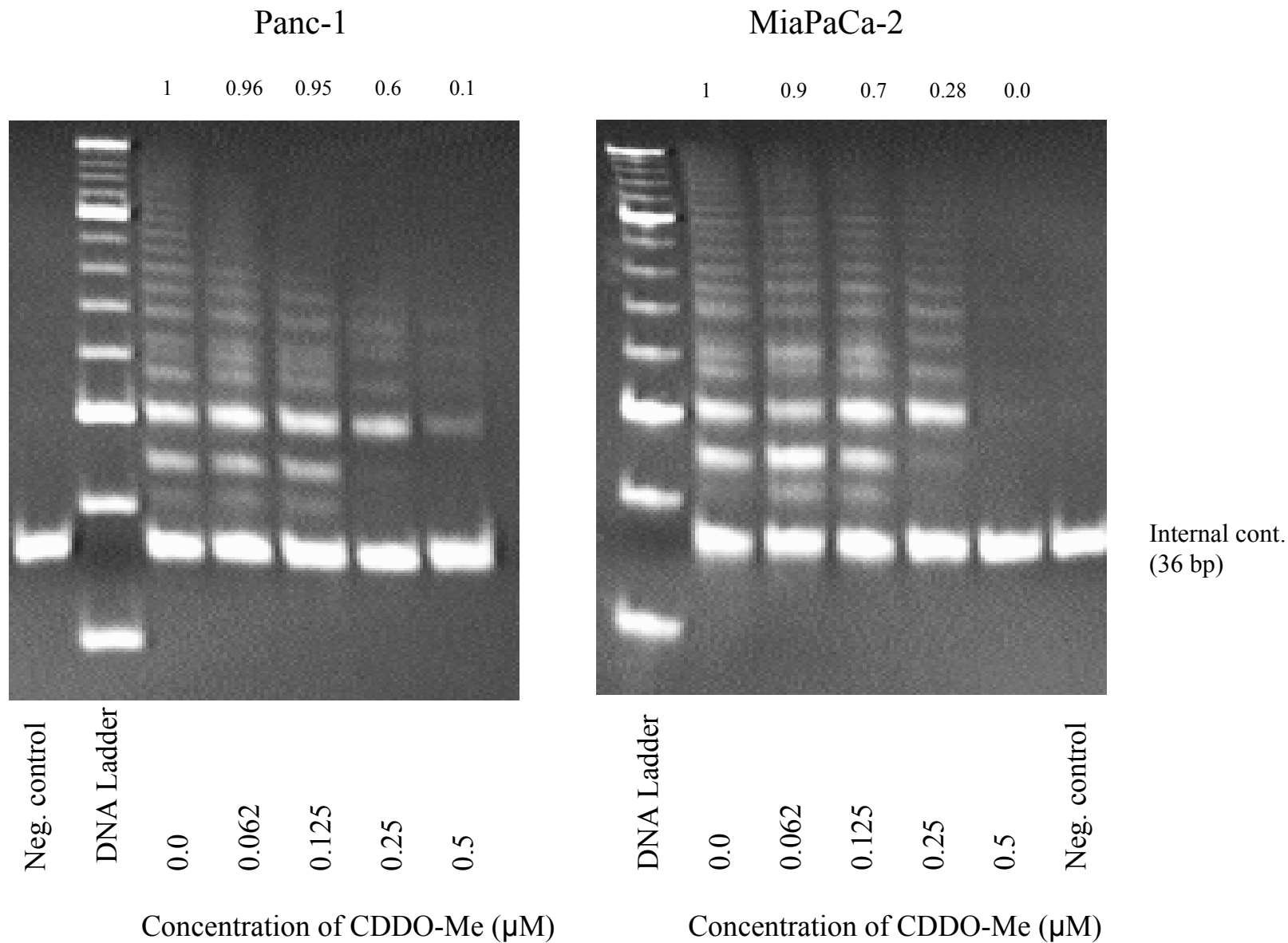

**Fig. S2.** Data showing effect of CDDO-Me on telomerase activity measured by PCR-based TRAP assay.

Supplement: Fig. S2 [file NIHMS613814-supplement-Fig__S2.pdf]
